# Translocator Protein 18 kDa (TSPO) as a Novel Therapeutic Target for Chronic Pain

**DOI:** 10.1155/2022/8057854

**Published:** 2022-08-29

**Authors:** Jie Liu, Jingyao Huang, Zhenjiang Zhang, Rui Zhang, Zhihao Zhang, Yongxin Liu, Baoyu Ma

**Affiliations:** ^1^Shandong Provincial Medicine and Health Key Laboratory of Clinical Anesthesia, School of Anesthesiology, Weifang Medical University, Weifang, China; ^2^Department of Thoracic Surgery, Weifang People's Hospital, Weifang, China

## Abstract

Chronic pain is an enormous modern public health problem, with significant numbers of people debilitated by chronic pain from a variety of etiologies. Translocator protein 18 kDa (TSPO) was discovered in 1977 as a peripheral benzodiazepine receptor. It is a five transmembrane domain protein, mainly localized in the outer mitochondrial membrane. Recent and increasing studies have found changes in TSPO and its ligands in various chronic pain models. Reversing their expressions has been shown to alleviate chronic pain in these models, illustrating the effects of TSPO and its ligands. Herein, we review recent evidence and the mechanisms of TSPO in the development of chronic pain associated with peripheral nerve injury, spinal cord injury, cancer, and inflammatory responses. The cumulative evidence indicates that TSPO-based therapy may become an alternative strategy for treating chronic pain.

## 1. Introduction

Chronic pain is a major public health issue, which seriously affects patients' quality of life and aggravates economic burdens on society. According to the International Association for the Study of Pain (IASP), chronic pain can be categorized as chronic primary pain and chronic secondary pain, (e.g., chronic cancer-related pain, chronic postoperative or posttraumatic pain, and chronic neuropathic pain) [1]. Opioids are powerful analgesics that are commonly used clinically. However, opioids can produce significant side effects, including respiratory depression, mental clouding, physical dependence, constipation, nausea, and vomiting [2, 3]. Despite increasing investigations into chronic pain mechanisms and therapeutic strategies [4, 5], current therapies for chronic pain management are severely insufficient or, as in the case of opioids, limited by serious side effects [6]. Therefore, exploring novel and effective analgesics for chronic pain treatment is crucial.

The translocator protein 18 kDa (TSPO) was discovered in 1977 as a peripheral benzodiazepine receptor [7]. This mitochondrial protein consists of 169 amino acids with five transmembrane domains [8], which is mainly localized in the outer mitochondrial membrane (OMM) [9]. TSPO can interact with specific mitochondrial proteins, like voltage-dependent anion channel (VDAC) and anion nucleotide translocator (ANT) [10–12], which are involved in the composition of mitochondrial permeability translocation pores and regulate development of physiological and pathological conditions [13], suggesting the key role of TSPO in cellular functions related to mitochondria. TSPO was initially designated as a peripheral-type benzodiazepine receptor, which is the binding site for benzodiazepines used to treat patients with anxiety, convulsions, or insomnia [7]. However, further research has shown that TSPO is widely expressed in most organs within the body, including the secretory and glandular tissues, kidney, heart, liver, and brain [8, 14, 15]. Its widespread distribution is consistent with its diverse physiological functions, including membrane biogenesis, heme biosynthesis, redox balance, bioenergetics, cell proliferation, apoptosis, immunomodulation, and cholesterol binding and transport [8, 9, 16–20]. In the nervous system, TSPO has been investigated as a biomarker for various neuropathologies [21]. Recently, TSPO and its ligands have been found to be effective in neurodegeneration [22], neuroinflammation [23], and neuropathic pain [24]. These studies highlight the potential uses of TSPO and its ligands for neuroprotection, limiting neuroinflammation, promoting regeneration, and treating nervous system dysfunctions. Herein, we review the current evidence for the role of TSPO and its ligands in the development of chronic pain, including neuropathic pain, cancer pain, and inflammatory pain (Table 1) [24–26].

## 2. Possible Mechanisms of TSPO in Pain Processing

The mechanisms of chronic pain development involve a variety of factors, among which neuroinflammation is an important mechanism resulting in this process [8]. Glia cells, through interactions with neurons via transmitters, cytokines, or chemokines, participate in neuroinflammation [45]. In the spinal nerve ligation (SNL) model, microglia release chemokine CXC motif ligand 1 (CXCL1); its transport to neurons results in neuroinflammation, promoting the development of chronic pain [27]. After chronic complete Freund's adjuvant (CFA) injections, interleukin-6 (IL-6) released from activated astrocytes can also activate neurons by promoting neuroinflammation, resulting in chronic pain [25]. These results indicated the role of glia in neuroinflammation and chronic pain.

TSPO is upregulated concomitant with microglial activation, and strong colocalization between TSPO upregulation and activated microglia has been found during neuroinflammation in various disorders, including neurodegeneration [46], peripheral nervous system lesions [47], and brain damage [48]. Hence, TSPO has served as a marker of microglial activation and neuroinflammation and as a predictor of chronic pain in disorders like fibromyalgia [49] and neuropathic pain [33]. Recently, TSPO was reported to participant in neuropathic pain [50], and it was shown that the TSPO-positive allosteric modulator koumine alleviates inflammation and neuropathic pain by modulating microglial activation and polarization, along with astrocyte activation [30, 50]. Wei et al. [24] found that the agonistic TSPO ligands Ro5-4864 and FGIN-1-27 decrease tumor necrosis factor-*α* (TNF-*α*) levels released by spinal activated astrocytes, alleviating neuroinflammation in a rat chronic pain model. Thus, in addition to serving as a marker for activated microglia and neuroinflammation, TSPO plays a protective role in neuroinflammation, which may inhibit chronic pain.

**Figure 1:**
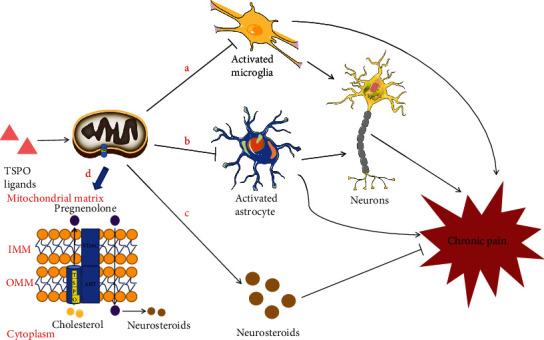
Mechanisms by which TSPO and its ligands inhibit chronic pain. (a, b) TSPO and its ligands inhibit microglial and astrocyte activation, thereby inhibiting activation-mediated central sensitization and chronic pain. (c) TSPO and its ligands inhibit chronic pain by mediating neurosteroid synthesis. (d) TSPO and its ligands promote cholesterol transport from the cytoplasm to the mitochondrial inner membrane, mediating progesterone synthesis and thus promoting neurosteroid synthesis. TSPO: translocator protein; IMM: inner mitochondrial membrane; OMM: outer mitochondrial membrane; VDAC: voltage-dependent anion channel; ANT: adenine nucleotide transporter.

TSPO may also participate in the development of chronic pain by mediating the synthesis of neurosteroids [51]. Neurosteroids play important roles in the structure and function of the nervous system [51], including inflammation, synaptic transmission, pain, consciousness, and cognition [52]. Numerous studies have shown that maintaining the normal structure and function of the nervous system is essential for preventing chronic pain development [11]. Neurosteroids are a class of steroid hormones that are widely distributed in the central and peripheral nervous systems [53]. TSPO transports cholesterol from the cytoplasm to the mitochondria, after which the cholesterol entering the mitochondria is catalyzed by a series of enzymes to produce steroid hormones [54]. This role of TSPO for steroid hormone synthesis is thus rate-limiting. Coronel et al. [34] found that expressions of TSPO and steroidogenic enzyme 5*α* reductase (I/II), which participate in steroid hormone synthesis [55], are significantly reduced and that progesterone injections reverse TSPO expression, in spinal cord injury (SCI). This demonstrated a correlation between TSPO and steroid hormone synthesis. Another group observed an increase in thalamic steroid hormone synthesis in the chronic phase of a rat pain model, which was accompanied by increased TSPO expression. Intrathalamic administration of steroid hormone, or AC-5216 (a TSPO agonist), reduced rats' pain symptoms; this effect was counteracted by administration of a TSPO inhibitor [56]. These studies suggest an inhibitory effect of steroid hormones on pain, and TSPO, as a rate-limiting enzyme for steroid hormone synthesis, may reduce pain by regulating neurosteroid synthesis (Figure 1).

## 3. The Role of TSPO in Chronic Pain

### 3.1. TSPO and Neuropathic Pain

The IASP defines neuropathic pain as pain caused by a lesion or disease of the somatosensory nervous system [57, 58]. Neuropathic pain is characterized by abnormal hypersensitivity to noxious stimuli (hyperalgesia) and abnormal pain responses to nonnoxious stimuli (allodynia) [59]. A-*δ* fiber and C-fiber primary afferent neurons in the peripheral nervous system can transport pain signals to the central nervous system. Peripheral nerve injury (PNI) can activate sensitized neurons in these fibers. The latter also produce continuous pain stimulation signals [60], which can then transport to the dorsal horn of the spinal cord, further promoting central sensitization. Various animal models have been used to explore the mechanisms of neuropathic pain in PNI, SCI, and diabetes [61]. A growing volume of recent evidence indicates that TSPO plays a critical role in neuropathic pain.

#### 3.1.1. TSPO and Peripheral Nerve Injury

Many studies have used animal models of PNI to explore novel strategies attenuating neuropathic pain and nerve regeneration. In early studies, TSPO improved peripheral nerve regeneration and functional recovery after nerve lesions [22, 28]. Daily intraperitoneal injections (i.p.) of etifoxine, an agonistic TSPO ligand, can promote axonal regeneration and improve functional recovery in rats with sciatic nerve injury (SNI) [22]. Another agonistic TSPO ligand, Ro5-4864, can substantially enhance adult facial motor neuron nerve regeneration and restore function after facial nerve axotomy [28]. In nerve stumps of SNI model rats, interleukin-1*β* (IL-1*β*) and IL-6 mRNA levels are significantly upregulated, while etifoxine treatment limits their increase in the proximal stumps [22]. These inflammatory cytokine changes indicate that TSPO ligands can influence inflammation in the peripheral nerve.

In recent years, investigators have focused on the role of neuroinflammation in neuropathic pain induced by PNI, many of whom have evaluated the influence of TSPO-mediated anti-inflammation in this condition. TSPO expression is significantly increased in a L5 SNL rat model [24], and a single intrathecal injection of TSPO agonist Ro5-4864 or FGIN-1-27 alleviates the established mechanical allodynia and thermal hyperalgesia. Subsequently, upregulated TSPO was decreased when neuropathic pain healed naturally followed by administration of these agonists. This phenomenon may explain that TSPO might promote recovery from a neuropathic pain state. Enzyme-linked immunosorbent assay work has revealed that increased TNF-*α*, a proinflammatory cytokine, in the spinal dorsal horn is suppressed after Ro5-4864 treatment. Further, early upregulation of TSPO in astrocytes of the spinal dorsal horn partly inhibit chemokine CXC motif ligand 1-chemokine CXC motif receptor 2- (CXCL1-CXCR2-) dependent astrocyte-to-neuron signaling and central sensitization, eliciting potent analgesic effects against chronic neuropathic pain induced by SNL [27]. These studies cumulatively suggest that TSPO agonists can inhibit neuroinflammation induced by proinflammatory cytokines or chemokines in a SNL model against chronic neuropathic pain.

**Figure 2:**
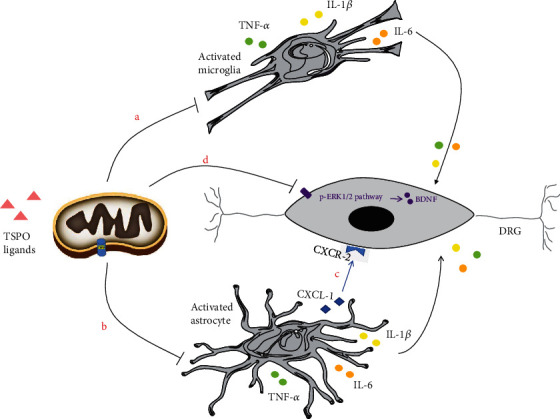
Molecular mechanisms by which TSPO and its ligands inhibit chronic pain induced by peripheral nerve injury. (a, b) TSPO and its ligands inhibit microglia and astrocytes to secrete inflammatory factors TNF-*α*, IL-1*β*, and IL-6. These inflammatory factors can cause neuroinflammation leading to chronic pain. (c) TSPO and its ligands inhibit CXCL1-CXCR2-dependent astrocyte-to-neuron signaling and central sensitization. (d) TSPO and its ligands inhibit the expression of the p-ERK1/2 signaling pathway and its downstream BDNF in DRG. BDNF is synthesized in DRG causing chronic pain by regulating neurotransmitter production. TNF-*α*: tumor necrosis factor-*α*; IL-1*β*: interleukin-1*β*; IL-6: interleukin-6; CXCL1: chemokine CXC motif ligand 1; CXCR2: chemokine CXC motif receptor 2; p-ERK1/2: phospho-extracellular signal-regulated kinase 1/2; BDNF: brain-derived neurotrophic factor; DRG: dorsal root ganglion.

Proinflammatory cytokines TNF-*α* and IL-1*β* participate in neuropathic pain in a chronic constriction injury (CCI) model [29]. Jin et al. [30] described TSPO's inhibitory effects on increased levels of these two proinflammatory cytokines and CCI-evoked microglial and astrocyte activation, in the spinal cord of a rat CCI model using koumine, a potential agonist. Further, TSPO was activated in neurons of the rat dorsal root ganglion (DRG) after spared nerve injury [31], and a single intrathecal injection of Ro5-4864 exerts remarkable analgesic effect in a SNI model. Western blotting shows that increased levels of phospho-extracellular signal-regulated kinase 1/2 (p-ERK1/2) and brain-derived neurotrophic factor (BDNF) in the DRG are inhibited after Ro5-4864 injection. Activation of the ERK1/2 signaling pathway and its downstream BDNF in DRG neurons also participate in the development of neuropathic pain after PNI via peripheral inflammation [32]. In that study, all increased levels of activated TSPO were suppressed or returned to normal after neuropathic pain healing, indicating that the role of TSPO upregulation may be to induce this recovery. The mechanism of its inhibition effect may be also involved in anti-neuroinflammation in PNI models (Figure 2).

#### 3.1.2. TSPO and Spinal Cord Injury

In addition to primary motor and sensory deficits, patients suffering from SCI may experience other debilitating consequences, including neuropathic pain [62]. Neuropathic pain after SCI (SCI-associated neuropathic pain) remains difficult to treat because its mechanism is yet unclear. Microglia- or astrocyte-mediated neuroinflammation in the dorsal horn participates in the development and maintenance of neuropathic pain after SCI [63]. Many studies have used PET imaging of PK11195, which binds to TSPO, to assess the role of glial activation, including that in neuroinflammatory processes, in neuropathic pain patients [33]. Recent reports suggest that TSPO can improve chronic SCI-induced neuropathic pain [34, 35]. Li et al. found that intragastric administration of ZBD-2 (N-benzyl-N-ethyl-2-(7,8-dihydro-7-benzyl-8-oxo-2-phenyl-9H-purin-9-yl) acetamide), an agonistic TSPO ligand, markedly reduces mechanically induced allodynia and thermal hyperalgesia in a SCI model [35]. Immunohistochemistry and western blot data suggest reversed effects of ZBD-2 on chronic activation of microglia and astrocytes after SCI, further explaining the relation between TSPO and neuroinflammation.

Liu et al. [34] also found an effect of TSPO in SCI through another mechanism: the steroid hormone progesterone. This hormone activated TSPO, downregulating mechanical and thermal allodynia in a SCI model. Progesterone administration can also reduce spinal expression of proinflammatory enzymes and cytokines in chronic neuropathic pain [36, 37], suggesting a potential role of progesterone in TSPO and neuroinflammation. These findings suggest that TSPO and its ligands may be potential targets for the treatment of SCI-associated neuropathic pain.

### 3.2. TSPO and Cancer Pain

Chronic pain is among the most common, burdensome, and feared cancer complications [64]. Many patients with advanced cancer experience severe pain, for which adequate analgesia strategies, without unacceptable side effects, remain a clinical challenge [65, 66]. Despite concomitant neuropathic and inflammatory pain, cancer pain's additional distinctive characteristics include metastatic cancer-induced bone pain [67]. Increased attention has been paid to investigating the role of neuroinflammation in cancer pain [68]. Herein, we focus on the anti-inflammation effects of TSPO in chronic pain from bone metastasis. TSPO level is increased in both astrocytes and microglia of a rat model for bone cancer pain (BCP) [26]. Further, a single intrathecal administration of midazolam (MZL), an agonistic TSPO ligand [38], can inhibit thermal hyperalgesia in BCP rats via spinal activation of TSPO. Increased expression of the inflammatory cytokine IL-6 is decreased after MZL injection. IL-6 is considered a trigger for the initiation and maintenance of chronic pain [38]. In the BCP model, IL-6 expression increased in the DRG and spinal cord [26, 39]. Remarkably, Fang et al. attenuated bone cancer-induced hyperalgesia in BCP rats by inhibiting the IL-6/sIL-6R trans-signaling pathway, implicating IL-6 in the development of BCP [39]. Increased IL-6 expression was decreased after MZL injection, indicating that TSPO may play a protective role in bone cancer-induced inflammation. Furthermore, the spinal MAPK ERK pathway, which participates in neuroinflammation [40], was inhibited in MZL-attenuated thermal hyperalgesia in BCP rats. These changes in two inflammation mediators indicate that glial TSPO may be a potential target for bone cancer-induced neuroinflammation.

### 3.3. TSPO and Inflammatory Pain

Inflammatory pain is the most important clinical symptom of inflammatory diseases. It can severely impact the quality of life among patient, suffering from osteoarthritis (OA), rheumatoid arthritis, and other diseases [25]. Prolonged inflammatory pain can lead to activation of spinal microglia and astrocytes [69]. Investigators have explored whether increased TSPO can serve as a marker of nervous system activation in an OA pain model, for use in predicting development of chronic pain using novel PBR/TSPO imaging agents [70–72]. In recent animal inflammatory pain models, TSPO was identified as playing anti-inflammation and antinociception roles in chronic pain. To build a CFA-induced monoarthritic model, rats were injected with CFA into the right tibiotarsal joint [25]; intra-articular CFA injection reliably induced thermal hyperalgesia and mechanical allodynia, and spinal TSPO expression was increased in neurons, astrocytes, and microglia, implying activated nervous system activity. Furthermore, intrathecal TSPO agonist (Ro5-4864) administration dose-dependently inhibited CFA-induced mechanical allodynia and thermal hyperalgesia. A similar anti-inflammatory effect was detected in formalin-induced chronic pain and ATP-induced NLRP3 inflammasome activation [41, 44]. A previous study also found that treatment with a TSPO ligand can downregulate proinflammatory mediator IL-1*β* expression and activate the NLRP3 inflammasome [41]. Spinal IL-1*β* can play an important role in formalin- and CFA-induced inflammatory pain [42, 43]. These cumulative findings suggest that TSPO may play an anti-inflammatory effect by mediating the release of these proinflammation cytokines, exerting part of their antinociceptive effects on inflammatory pain.

## 4. Conclusions

In this review, we reviewed the relations between TSPO and chronic pain. Cumulative findings have clarified that TSPO plays a vital role in alleviating the initiation and maintenance of chronic pain, including neuropathic pain, inflammatory pain, and cancer pain. Although TSPO expression is increased under inflammatory conditions [73, 74], it acts as a negative regulator of inflammation [75]. In the studies described herein, expression of proinflammation factors like IL-1*β*, IL-6, and CXCL1 is decreased after overexpression or activation of TSPO, further validating the role of TSPO in the inhibition of the neuroinflammatory response. Although the mechanisms of TSPO's effective role in nerve diseases has been validated through clinical trials [76], the clinical effects of TSPO as an agent for alleviating chronic pain have not yet been explored. However, the preclinical TSPO studies described herein provide novel insight that may facilitate drug development to advance pain therapies. Further clinical trials will now be needed to explore the effects of TSPO treatment for pain alleviation.

## Figures and Tables

**Table 1:** Role of TSPO and its ligands in chronic pain.

Model	TSPO/ligand	Mechanism	Reference(s)
SNL	TSPO	Inhibit CXCL1-CXCR2-dependent astrocyte-to-neuron signaling and central sensitization	[24, 27]
	Ro5-4864	Downregulate expression of TNF-*α*; alleviate mechanical allodynia and thermal hyperalgesia	[27, 28]
	FGIN-1-27	Downregulate expression of TNF-*α*, alleviate mechanical allodynia and thermal hyperalgesia	[27, 28]
CCI	Koumine	Downregulate the expression of TNF-*α* and IL-1*β*; inhibit microglia and astrocyte activation	[29, 30]
SNI	Ro5-4864	Downregulate the expression of p-ERK1/2 and BDNF in DRG; improve axonal regeneration and functional recovery	[31, 32]
SCI	PK11195	Assess the role of glial activation	[33]
	ZBD-2	Inhibit chronic activation of microglia and astrocytes; alleviate mechanical allodynia and thermal hyperalgesia	[34, 35]
	Progesterone	Reduce spinal expression of proinflammatory enzymes and cytokines	[36, 37]
CIBP	MZL	Inhibit the IL-6/sIL-6R trans-signaling pathway and MAPK ERK pathway; alleviate thermal hyperalgesia	[26, 38] [39, 40]
CFA-induced	TSPO	Downregulate proinflammatory mediator IL-1*β* expression	[41–43]
Monoarthritic model	Ro5-4864	Alleviate mechanical allodynia and thermal hyperalgesia	[41, 44]

SNL: spinal nerve ligation; CCI: chronic constriction injury; SNI: sciatic nerve injury; SCI: spinal cord injury; CIBP: cancer-induced bone pain; CFA: Complete Freund's adjuvant; TSPO: translocator protein; ZBD-2: N-benzyl-N-ethyl-2-(7,8-dihydro-7-benzyl-8-oxo-2-phenyl-9H-purin-9-yl) acetamide; MZL: midazolam; CXCL1: chemokine CXC motif ligand 1; CXCR2: chemokine CXC motif receptor 2; TNF-*α*: tumor necrosis factor-*α*; BDNF: brain-derived neurotrophic factor; DRG: dorsal root ganglion.
